# Preparation and Performance of Poly(Butylene Succinate) (PBS) Composites Reinforced with Taxus Residue and Compatibilized with Branched PBS

**DOI:** 10.3390/polym17192597

**Published:** 2025-09-25

**Authors:** Shiwanyi Chen, Shufeng Li, Bing Wang, Chen Chen, Liuchun Zheng

**Affiliations:** 1 State Key Laboratory of Separation Membranes and Membrane Processes, School of Textile Science and Engineering, Tiangong University, Tianjin 300387, China2220010029@tiangong.edu.cn (B.W.); 2 School of Materials Science and Engineering, Shanghai University of Engineering Science, Shanghai 201620, China; qpchenchen@126.com; 3 School of Chemical Engineering and Technology, Xinjiang University, Urumqi 830017, China

**Keywords:** poly(butylene succinate), taxus residue, branching, biodegradable

## Abstract

In response to the escalating plastic pollution crisis, the development of high-performance biodegradable materials is critical. Poly(butylene succinate) (PBS) is an important biodegradable polymer as it possesses excellent biodegradability and processability. But it suffers from limitations such as low mechanical strength, poor thermal stability, and high production costs. In this study, taxus residue (TF), a waste by-product, was utilized as a reinforcing filler to reduce PBS costs while enhancing its overall performance. To address the interfacial incompatibility between TF and PBS, branched PBS (T-PBS) was introduced as a compatibilizer. The TF was surface-modified via alkali treatment and silane coupling (KH550), and a series of PBS/TF/T-PBS composites with varying T-PBS viscosity grades were prepared by melt blending. The compatibilization mechanism of T-PBS and its influence on the composite structure, crystallization behavior, thermal stability, rheological, and mechanical properties were systematically investigated. Results show that the branched structure significantly enhanced T-PBS melt strength and reactivity. The introduction of T-PBS effectively improved interfacial compatibility between TF and PBS matrix, reducing phase separation and interfacial defects. Compared to uncompatibilized PBS/TF composites, those with appropriately viscous T-PBS exhibited improved tensile strength (increased by 19.7%) and elongation at break (increased by 78.8%), while flexural strength was also maintained at an enhanced level. The branched points acted as nucleating agents, increasing the onset temperature and degree of crystallinity. In the high-temperature region, the synergistic barrier effect from TF and char residue improved thermal stability (*T*_85%_ reached 408.19 °C). Rheological analysis revealed enhanced viscosity and elasticity of the system. This study provides a promising strategy and theoretical foundation for the high-value utilization of taxus waste and the development of high-performance biodegradable PBS-based composites.

## 1. Introduction

With the rapid advancement of polymer science, plastics have become increasingly prevalent in everyday consumer products. They can be divided into biodegradable plastics and non-biodegradable plastics. However, approximately 60% of non-biodegradable plastic wastes are disposed of through landfilling or incineration [1], leading to severe resource depletion and environmental challenges [2,3]. As a result, biodegradable plastics have emerged as a critical research focus within the field of materials science [4,5]. Poly(butylene succinate) (PBS) is a biodegradable aliphatic polyester that possesses excellent biocompatibility and has been widely investigated for use in biomedical, packaging, and textile applications [6,7]. Despite its favorable mechanical properties, PBS suffers from limitations such as relatively low tensile strength, slow crystallization rate, poor thermal stability, and comparatively high production costs. These drawbacks restrict its broader application in high-performance engineering fields such as automotive manufacturing and construction [8,9,10,11]. To address the high cost and performance limitations of PBS, recent studies have explored the incorporation of various fillers—including natural plant fibers, plasticized starch, and calcium carbonate [12]—to reduce material costs while simultaneously improving composite performance [13]. Among these, natural fibers have garnered significant attention due to their biodegradability, renewability, and environmental friendliness.

In recent years, natural fiber-reinforced composites have experienced rapid growth and widespread application [14]. A variety of agricultural and industrial residues, such as coconut shells [15], hemp fibers [16], straw fibers [17], almond shells [18], and lignin [19], have been explored as reinforcing agents for PBS-based matrices. However, a critical issue remains: poor interfacial compatibility between hydrophilic natural fibers and hydrophobic polymer matrices often leads to inefficient stress transfer [20]. To overcome this challenge, many researchers have introduced compatibilizers to enhance interfacial adhesion between the fiber and matrix. For example, maleic anhydride (MAH) has been widely used as a grafting agent to promote interfacial reactions between cellulose-based fillers and polymer chains [21], significantly improving mechanical properties and enabling scalable composite production [22]. Similarly, isocyanate treatments have been employed in industrial applications [23], where cellulose surfaces are readily modified via chain extension or crosslinking reactions [24]. However, such compatibilizers are typically non-biodegradable and environmentally unfriendly, potentially compromising the sustainability of the final composites.

In this study, waste residues from taxus (TF), a rare and renowned natural anticancer plant rich in paclitaxel [25], were selected as reinforcement for PBS composites. Excessive harvesting of taxus has led to large amounts of waste biomass, raising environmental concerns [26]. TF exhibits a unique microstructure and favorable mechanical properties, including thick cell walls and high cellulose content, resulting in superior strength and dimensional stability [27]. Utilizing TF as reinforcement not only enhances the mechanical and thermal performance of PBS composites but may also impart certain bioactivities, thus broadening their applicability in biomedical fields and promoting value-added utilization of biomass waste. To further improve interfacial compatibility between TF and the PBS matrix, a branched form of PBS (T-PBS) was synthesized and introduced as a compatibilizer. The branching modification enhances the melt strength and reactivity of PBS [28]. In this study, trimethylolpropane (TMP) was employed as a triol branching agent to generate branched structures while preserving terminal hydroxyl groups capable of interacting with fiber surfaces. Compared to linear PBS, the branched architecture facilitates more uniform stress distribution, reduces material brittleness, and suppresses crack propagation, thereby improving elongation at break without compromising strength [29]. Moreover, the polar side chains or terminal groups of T-PBS can interact with reactive functional groups (e.g., lignin and polysaccharides) present on the TF surface through hydrogen bonding or chemical bonding, thus reducing phase separation. The introduction of branched structures not only mitigates interfacial defects but also enhances stress transfer efficiency, resulting in improved compatibility between the PBS matrix and TF reinforcement, and ultimately leading to better mechanical performance.

In summary, this study explores the compatibilization mechanism of T-PBS in PBS/TF composites and provides a theoretical basis for the development of high-performance, fully biodegradable composite materials.

## 2. Experimental Section

### 2.1. Materials

Succinic acid (SA, 99%) and 1,4-butanediol (BDO, 99%) were purchased from J&K Chemical (Beijing, China). Citric acid, tartaric acid, trimethylolpropane (TMP), tetrachloroethane, magnesium acetate [Mg(Ac)_2_], titanium glycolate, phenol, and other reagents were obtained from Aladdin Reagent Co., Ltd. (Shanghai, China). Taxus residue (TF), γ-aminopropyltriethoxysilane (KH550) (Shanghai, China), and sodium hydroxide (NaOH) (Shanghai, China) were also used. All chemicals were used as received without further purification.

### 2.2. Synthesis of PBS and T-PBS

PBS and branched PBS (T-PBS) were synthesized via a two-step process involving esterification followed by polycondensation. Under nitrogen (N_2_) atmosphere, BDO, SA, and TMP (in molar percentage based on SA) were added to a reaction vessel with continuous stirring. The detailed reaction factors are provided in Table 1. Magnesium acetate (0.12 wt% relative to the theoretical product) was added as an esterification catalyst, and the temperature was raised to 180 °C. Upon reaching the theoretical water content, the temperature was increased to 230 °C. Titanium glycolate (0.1 wt% of the theoretical product) was then introduced as a polycondensation catalyst, and the pressure was reduced to 50 Pa to initiate polycondensation. By adjusting the polycondensation time, T-PBS samples with different viscosities were obtained. To prepare pure PBS, TMP does not need to be added, and all other preparation conditions are the same.

**Scheme 1:**
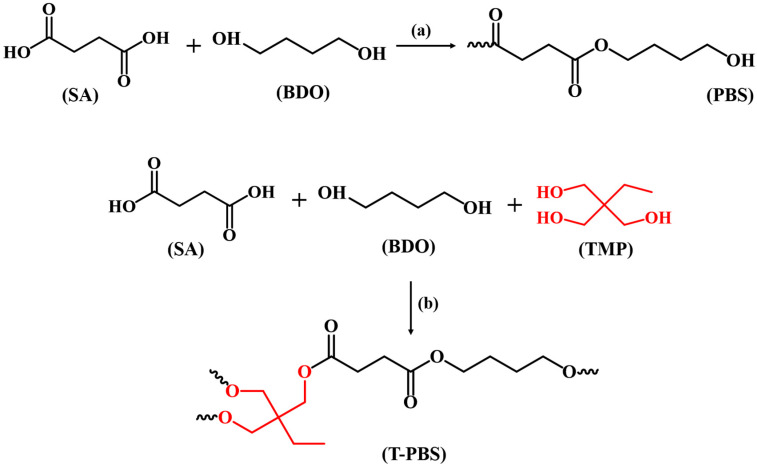
Synthesis route of PBS and T-PBS.

Here, T-PBS refers to PBS containing branched structures; the numerical suffix in T-PBS_x_ denotes an increase in polycondensation time. The overall synthetic route is illustrated in Scheme 1. And the addition ratios of SA, BDO, and TMP here represent the optimal proportions determined in our preliminary experiments.

### 2.3. Pretreatment of Taxus Residue (TF)

**Scheme 2:**
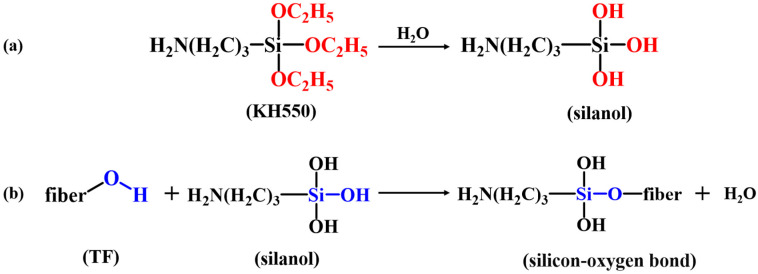
Mechanism of modification of TF by silane coupling agent (KH550): (**a**) hydrolysis of siloxane; (**b**) reaction of silanol with hydroxyl.

Taxus residue (TF) were first sieved and vacuum-dried. Dried TF was then added to a 5 wt% NaOH aqueous solution, solid–liquid ratio of 1:8 and stirred at 45 °C for 90 min. The treated TF was filtered and vacuum-dried. Then, KH550 silane coupling agent, at 2 wt% relative to the mass of TF, was dissolved in a 90% ethanol–water solution and stirred at room temperature for 2 h to ensure uniform hydrolysis. Subsequently, alkali-treated TF was added to the KH550 solution at a solid–liquid ratio of 1:10 and further stirred magnetically for 4 h. The modified TF was then vacuum-filtered, dried to constant weight, ground, and sieved to obtain silane-functionalized TF [30,31]. The silanization mechanism using KH550 is illustrated in Scheme 2.

### 2.4. Preparation of PBS/TF/T-PBS Composites

The dried TF, PBS, and T-PBS were blended in a laboratory internal mixer according to the proportions listed in Table 2 (ratios previously optimized through preliminary trials). The blending was carried out at 150 °C with a rotor speed of 40 rpm for 10 min. The obtained blend was crushed into pellets and then injection-molded into tensile specimens. Standard test specimens were subsequently prepared using a universal sample cutting machine.

### 2.5. Characterization

#### 2.5.1. Injection Molding

The sample was dried at 70 °C for 8 h. A barrel temperature of 130 °C and a mold temperature of 30 °C, injection time of 10 s, and holding time of 5 s were used. The composite particles were processed into dumbbell-shaped specimens (75.0 × 5.0 × 2.0 mm) according to ISO 527 [32] and rectangular specimens (80.0 × 10.0 × 4.0 mm) according to ISO 178 [32].

#### 2.5.2. Intrinsic Viscosity

The intrinsic viscosity of polyester is measured using an Ubbelohde viscometer at 25 °C ± 0.1 °C by dissolving 0.125 g of polyester in 25 mL of a mixed solvent of phenol and tetrachloroethane (1:1 m:m). A solution of 0.5 g/dL is prepared, and each sample is tested five times to obtain the average value.

(1) $$\eta_{r}=\frac{t_{1}}{t_{0}}$$

(2) $$\eta_{sp}=\frac{t_{1}-t_{0}}{t_{0}}$$

(3) $$\left[\eta \right]=\frac{\sqrt{2(\eta_{sp}-\ln\eta_{r})}}{C}$$

where $\eta_{r}$ is the relative viscosity, $\eta_{sp}$ is the specific viscosity, C is the concentration, and t_1_ and t_0_ are the flow times of the solution and pure solvent, respectively.

#### 2.5.3. ^1^H NMR Analysis

^1^H nuclear magnetic resonance (NMR) spectra were recorded on a Bruker AVIII 400 spectrometer (400 MHz, Bruker BioSpin, Co., Ettlingen, Germany) at 25 °C, using CDCl_3_ as the solvent and tetramethylsilane (TMS) as the internal standard.

#### 2.5.4. ATR-FTIR Analysis

ATR-FTIR spectra were collected on a Bruker ALPHA spectrometer equipped with an attenuated total reflectance module. Each sample was scanned 32 times at a resolution of 4 cm^−1^ over a wavenumber range of 4000–500 cm^−1^.

#### 2.5.5. Wide-Angle X-Ray Diffraction (XRD)

XRD patterns were recorded on a Bruker AXS D8 Advance diffractometer using Cu Kα radiation (λ = 0.154 nm). Scans were conducted at room temperature over a 2θ range of 10–45°, at a scan rate of 2°/min. At least five specimens per sample were tested, and average values were reported.

The crystallinity (*X*_c*_) was calculated from Equation (4) after fitting the diffraction images and merging the peaks, where S_a_ and S_c_ represent the areas of the crystalline and amorphous peaks, respectively.

(4) $$X_{c^{*}}\%=\frac{S_{c}}{S_{c}-S_{a}}\times 100\%$$

#### 2.5.6. Differential Scanning Calorimetry (DSC)

Thermal transitions of PBS and its composites were analyzed using a DSC instrument (Q5800, PerkinElmer Co., Waltham, MA, USA). Approximately 6.0 mg of each sample was sealed in an aluminum pan and heated to 180 °C at a rate of 10 °C/min under a nitrogen atmosphere. After maintaining this temperature for 5 min to eliminate thermal history, samples were cooled to −60 °C at the same rate, and then reheated to 180 °C at 10 °C/min. Heating and cooling curves were recorded to determine the melting point (*T*_m_) and entropy of melting *(*∆*H*_m_), cold crystallization temperature (*T*_c_), and enthalpy of crystallization (∆*H*_c_).

The crystallinity (*X*_c_) was calculated using the following equation:

(5) $$X_{c}\%=\frac{∆H_{m}-∆H_{c}}{∆H_{m}^{\theta }}\times 100\%$$

$H_{m}^{\theta }$∆ = 110.3 J/g represents the enthalpy of 100% crystalline PBS [33].

#### 2.5.7. Thermogravimetric Analysis (TGA)

Thermal stability was assessed using a TGA instrument (Pyris, PerkinElmer, Co., USA) under nitrogen atmosphere. Samples were heated from 40 °C to 800 °C at a constant rate of 10 °C/min.

#### 2.5.8. Rheological Analysis

Rheological measurements were carried out on an MCR 302 rotational rheometer using parallel plates with a diameter of 25.0 mm and a gap of 1.0 mm. Frequency sweep tests were performed at 170 °C over a range of 0.01–100 Hz at a constant strain of 1%. Disk-shaped specimens (25 mm diameter, 1.1 mm thickness) were prepared using a HAAKE MiniJet II injection molding machine and preheated at 100 °C before testing.

#### 2.5.9. Mechanical Testing

Tensile, flexural, and impact properties were evaluated. Prior to testing, all samples were dried at 70 °C under vacuum for 24 h. **Tensile tests** were conducted on dumbbell-shaped specimens (75.0 × 5.0 × 2.0 mm) using an Instron 1122 universal testing machine at a crosshead speed of 50 mm/min, in accordance with ISO 527. **Flexural tests** were performed on rectangular specimens (80.0 × 10.0 × 4.0 mm) using the same equipment, following ISO 178. **Impact tests** were conducted on notched samples (80.0 × 10.0 × 4.0 mm) using a pendulum impact tester, in accordance with ISO 179 [32]. All specimens were prepared by injection molding at 175 °C under a pressure of 800 bar. At least five specimens per sample were tested, and average values were reported.

#### 2.5.10. Scanning Electron Microscopy (SEM)

Fracture surfaces of the impact-tested specimens were gold-sputtered and observed using SEM at an accelerating voltage of 10 kV to investigate the microstructural morphology.

## 3. Results and Discussion

### 3.1. Characterization of PBS and T-PBS

**Figure 1:**
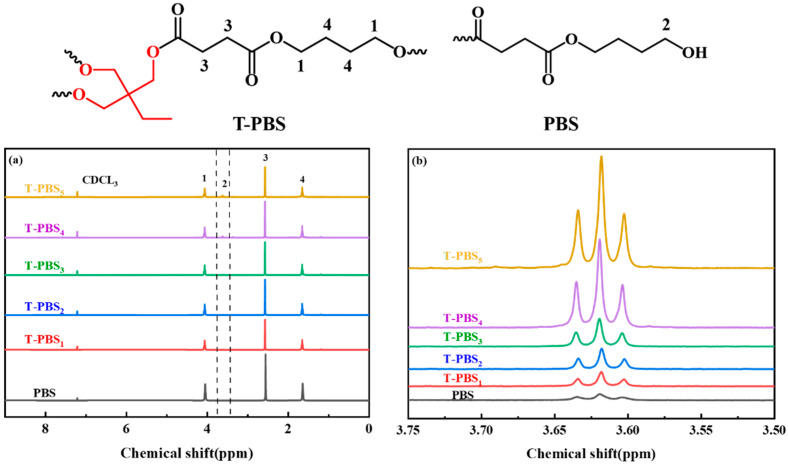
^1^H-NMR spectra of (**a**) PBS and T-PBS and (**b**) partial enlarged detail.

The chemical structures of PBS and T-PBS were characterized via proton nuclear magnetic resonance (^1^H NMR) spectroscopy, and the results are presented in Figure 1. As illustrated in Figure 1a, both PBS and T-PBS exhibited similar characteristic signals at 4.1 ppm (δH1), 3.5–3.7 ppm (δH2), 2.6 ppm (δH3), and 1.7 ppm (δH4), corresponding to the main chain protons [34]. A distinct triplet signal appeared at 3.63 ppm exclusively in T-PBS, which was attributed to the methylene protons in the terminal hydroxyl group (-(CH_2_)_3_CH_2_OH), confirming the presence of hydroxyl-terminated side chains introduced by the branching agent. In contrast, linear PBS displayed only weak terminal group signals due to its high molecular weight and low terminal hydroxyl content. The incorporation of the branching agent led to an increase in terminal hydroxyl concentration, thereby significantly enhancing the intensity of the corresponding proton signals (particularly δH2), indicating the successful synthesis of the branched structure.

As illustrated in Figure 1b, the introduction of TMP as a branching agent, along with the increase in T-PBS viscosity, resulted in a greater degree of branching, thereby improving the effectiveness of the branched architecture. The intrinsic viscosities of PBS and various T-PBS samples are summarized in Table 3. It is worth noting that prolonged polycondensation times following the addition of the branching agent may lead to higher viscosities and potentially induce the formation of crosslinked structures, which can adversely affect the processability of the polymer. According to Table 3, the intrinsic viscosity of T-PBS increased progressively with polycondensation time. T-PBS_5_ exhibited the highest viscosity (1.85 dL/g) without evidence of crosslinking, suggesting that even at elevated viscosity levels, the branched structure remained within a processable range.

**Figure 2:**
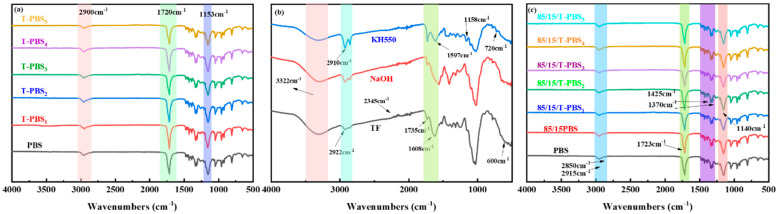
FT-IR spectrum of (**a**) PBS and T-PBS, (**b**) TF before and after NaOH and KH550 modification, (**c**) PBS-based composites.

The chemical structures of PBS, branched PBS (T-PBS), taxus residues (TF) before and after NaOH and KH550 modification, and the PBS-based composites were further investigated using attenuated total reflectance Fourier transform infrared (ATR-FTIR) spectroscopy. In Figure 2a, the ATR-FTIR spectra of PBS and T-PBS exhibited characteristic stretching and bending vibrations of C–H bonds at 2900 cm^−1^ and 1153 cm^−1^, respectively. A strong absorption peak at 1720 cm^−1^ corresponded to the stretching vibration of the carbonyl (C=O) group in ester linkages.

Figure 2b illustrates the ATR-FTIR spectra of TF before and after NaOH and KH550 modification. The untreated TF showed a broad absorption band between 3000 and 3700 cm^−1^, attributable to the stretching vibrations of hydroxyl (–OH) groups present in cellulose and lignin. The peak near 2922 cm^−1^ was assigned to C–H stretching in methyl and methylene groups of lignin and wax components [35]. An absorption peak at 2345 cm^−1^ was related to the stretching vibration of C≡C bonds in waxy constituents [36]. The band at 1735 cm^−1^ was ascribed to C=O stretching of hemicellulose, while the peak at 1608 cm^−1^ corresponded to C=O stretching of carbonyl groups in hemicellulose and pectin [37]. Additionally, the band near 600 cm^−1^ was attributed to out-of-plane C–H bending of aromatic rings in lignin. Following alkaline treatment, no new peaks appeared in the spectrum, but noticeable changes in the intensities of several characteristic bands were observed. Specifically, the hydroxyl peak at 3322 cm^−1^ became slightly more intense, which was attributed to the removal of surface impurities and increased exposure of hydroxyl groups on TF fibers, thereby facilitating subsequent silane modification. The disappearance of the 2345 cm^−1^ peak indicated effective removal of waxy substances. The elimination of the 1735 cm^−1^ band and the attenuation of the 1608 cm^−1^ peak suggested the removal of pectin and partial hemicellulose, while the weakened signal near 600 cm^−1^ confirmed the reduction in lignin content. Collectively, these results demonstrate that alkali treatment effectively eliminated hemicellulose, portions of lignin and pectin, and wax impurities from TF, thereby increasing cellulose exposure and enhancing the potential for silane coupling with surface hydroxyl groups. After silanization, curve revealed new absorption peaks at 720 cm^−1^ and 1158 cm^−1^, corresponding to the characteristic vibrations of Si–O–Si and C–O–Si bonds, respectively. The band at 1158 cm^−1^ was specifically associated with the stretching of Si–O–cellulose linkages [38], confirming successful condensation between silanol groups from the coupling agent and hydroxyl groups on the TF surface. The reaction also led to a reduction in the intensity of the hydroxyl stretching band at 3322 cm^−1^, reflecting decreased –OH content, which would subsequently reduce the hydrophilicity of the composite. Moreover, the presence of methyl and methylene groups within the silane agent resulted in a slight enhancement of the C–H stretching peak at 2910 cm^−1^. A peak at 1597 cm^−1^ was assigned to N–H stretching from the amino group of the KH550 silane.

Figure 2c presents the ATR-FTIR spectra of the PBS-based composites. Peaks at 2915 cm^−1^ and 2850 cm^−1^ attributed to –CH_2_ stretching vibrations, while the peaks at 1723 cm^−1^ and 1140 cm^−1^ corresponded to the C=O and C–O–C stretching vibrations, confirming the presence of ester bonds. Due to the complex polysaccharide structure of taxus fibers and the overlap of their characteristic bands with those of PBS, the specific peaks of TF were largely obscured. Furthermore, the addition of T-PBS did not introduce new functional groups, and thus no new absorption peaks were observed in the infrared spectra of the composites.

### 3.2. Wide-Angle X-Ray Diffraction of PBS and PBS-Based Composites

**Figure 3:**
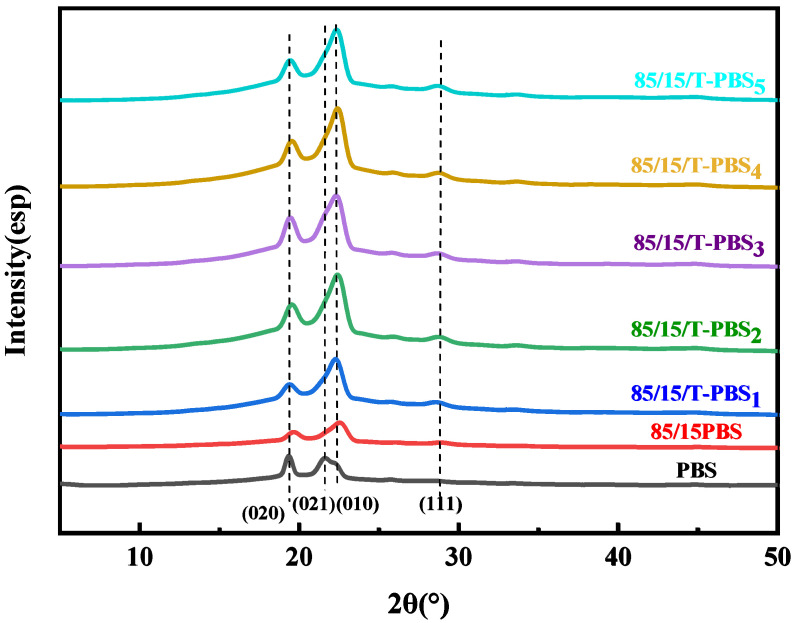
WAXD spectra of PBS and PBS-based composites.

To further investigate the relationship between PBS-based composites crystal structure and properties, the crystalline structure and crystallinity of the samples were characterized using Wide-Angle X-ray Diffraction (WAXD). As observed in Figure 3, characteristic diffraction peaks of PBS were detected at 2θ = 19.3°, 21.8°, 22.8° and 29.3°, corresponding to the (020), (021), (110) and (111) α-crystal planes [39], respectively. Compared with PBS, after adding TF, the intensity of diffraction peaks on the (010) crystal plane was enhanced. This is because TF contains a large number of polar functional groups and a semi-crystalline cellulose structure, which can provide rich heterogeneous nucleation sites for PBS molecular chains, thereby accelerating crystal formation and improving the crystallinity. However, other peaks did not show significant changes, indicating that the addition of TF did not alter the crystal morphology and crystal structure of the composite material [40]. Upon the introduction of T-PBS into the composites, the diffraction peaks corresponding to the (010) and (111) crystal planes gradually intensify, indicating that the introduction of branch structures promotes the formation of a more stable lamellar structures along these crystal planes. Studies on crystallization kinetics [41] have demonstrated that the presence of TMP within the polymer can act as a nucleating agent, accelerating the nucleation rate. Furthermore, branching is known to generate a greater number of chain ends, which can enhance chain mobility and increase crystallinity [42]. It can thus be seen that the synergistic effect of T-PBS and TF-induced nucleation jointly altered the crystalline morphology of PBS in the composite system and enhanced the orderliness of the system.

### 3.3. Thermal Properties

**Figure 4:**
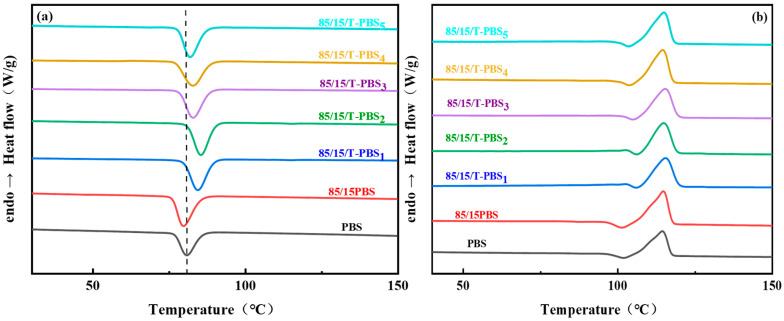
DSC spectra of PBS and composite materials (**a**) during the heating process and (**b**) cooling process.

The thermal properties of PBS and its composites were investigated using differential scanning calorimetry (DSC), as presented in Figure 4. The introduction of T-PBS exhibited no significant alteration in the thermal transition behavior of the composites; however, several subtle and systematic changes were observed. The presence of both crystallization and melting peaks in PBS and its composites confirms their semicrystalline nature. Notably, minor crystallization peaks appearing prior to melting are attributed to a melt–recrystallization–remelting mechanism [43], which is commonly associated with the formation of small and imperfect crystals during the cooling of melt crystallized at higher temperatures. According to the data summarized in Table 4, the melting temperature (*T*_m_) of the 85/15PBS composite is close to that of PBS, indicating that the incorporation of TF had no evident effect on the PBS crystalline structure, but it did introduce some interfacial imperfections. The crystallization temperature (*T*_c_) showed a slight decrease, suggesting that TF hindered the regular alignment of PBS molecular chains, thereby impeding chain mobility and reducing crystallization kinetics. Upon the addition of T-PBS, the *T*_c_ values of the ternary blends were consistently higher than those of PBS and 85/15PBS, indicating that the multiple branching points in T-PBS acted as effective heterogeneous nucleation centers, facilitating chain alignment and elevating the crystallization onset temperature [44].

The incorporation of TF led to significant increases in both melting enthalpy (∆*H*_m_) and crystallization enthalpy (∆*H*_c_), confirming that TF functioned as a crystallization nucleator and promoted the formation of crystalline domains. Furthermore, the addition of T-PBS resulted in fluctuations of ∆*H*_m_ and ∆*H*_c_ within the ranges of 51.91–56.20 J/g and 59.99–65.19 J/g, respectively, corresponding to crystallinity (*X*_c_) values between 47.1% and 50.9%, which remained higher than those of pure PBS. These results suggest that while the branched structure of T-PBS promotes nucleation, it may also introduce steric hindrance, restricting crystal growth to a certain extent. Nevertheless, a relatively high level of crystallinity was preserved across the ternary systems, which is in agreement with the results of the XRD tests. The optimized crystallization behavior of the PBS/TF/T-PBS composites can be attributed to improved interfacial interactions introduced by T-PBS, which enhanced phase compatibility and reduced phase separation, thereby increasing ∆*H*_c_. Conversely, excessive branching, as in 85/15/T-PBS_5_, is likely to result in chain entanglement, impeding crystallization as evidenced by a reduced ∆*H*_c_ of 59.99 J/g. These findings suggest the existence of an optimal T-PBS viscosity range for achieving superior overall crystallization performance.

**Figure 5:**
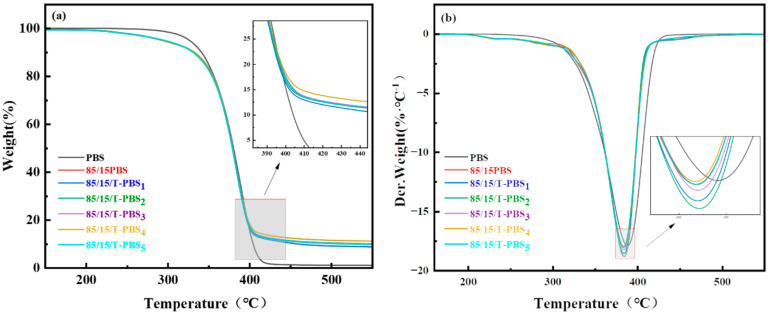
(**a**) TGA curves and (**b**) DTG curves of PBS and composite materials.

Thermogravimetric analysis (TGA) was employed to evaluate the thermal stability of PBS and its composites under nitrogen atmosphere, as shown in Figure 5. The initial decomposition temperature at 5% weight loss (*T*_5%_) for PBS was 325.65 °C, which was significantly higher than that of all the TF-containing composites. This was attributable to the presence of low thermal stability constituents in TF, such as hemicellulose and lignin. After incorporating T-PBS, the *T*_5%_ values of the composites remained nearly unchanged compared to the unbranched system. However, improvements were observed in both *T*_85%_ and *T*_max_. For instance, the *T*_85%_ of the 85/15/T-PBS_4_ sample reached 408.19 °C, slightly exceeding that of PBS (400.16 °C). It is attributed to the fact that branched structure increases the number of interactions between polymer chains, which can be chemically bonded or physically entangled, and they enhance the intermolecular interaction forces, making it more difficult for the molecular chains to move and become untangled when subjected to heat [45]. And in previous studies, Soatthiyanon N. et al. [46] selected salt-treated ramie fiber (KTH), extracted cellulose fiber (EC), and commercial cellulose fiber (CC) as renewable fillers to blend PBS. The PBS/KTH composite (*T*_max_ = 405.1 °C) exhibited the best thermal stability among the products tested, yet its performance remained worse than that of the 85/15/T-PBS_4_ composite developed in this study. In summary, the co-introduction of TF and T-PBS significantly enhanced the crystallization behavior and crystallinity of PBS-based composites. Simultaneously, the formation of a dense carbonaceous layer during high-temperature degradation conferred improved thermal stability, providing a robust thermal foundation for the application of PBS in high-performance bio-based composite materials.

### 3.4. Mechanical Properties

**Figure 6:**
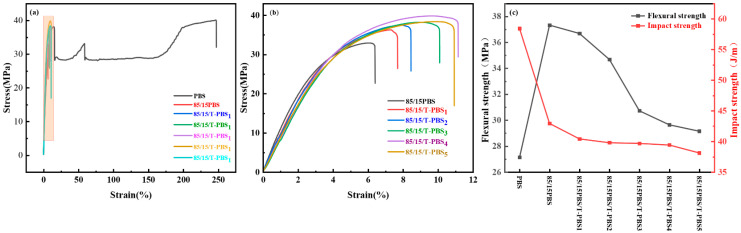
Mechanical properties of PBS and composite materials. (**a**) Tensile curves of PBS and PBS-based composites; (**b**) tensile curves within the range of 0–12%; (**c**) impact strength and flexural strength.

Table 5 summarizes the mechanical properties of PBS and its composites. Compared with PBS, the incorporation of TF led to a 37.5% increase in flexural strength in the 85/15PBS composite. This enhancement can be attributed to the intrinsic rigidity of the TF fibers, which contributed to improved stiffness of the composite. However, as shown in Figure 6a, yield strength and elongation at break exhibited noticeable decreases upon TF addition. This decline is primarily due to the poor compatibility between the cellulose and lignin components of natural plant fibers and the thermoplastic PBS matrix. Additionally, the presence of residual natural waxy substances on the fiber surfaces further weakens the interfacial adhesion and reduces the wettability of the PBS matrix on the fiber surface. Furthermore, TF tends to agglomerate within the polymer matrix, resulting in uneven dispersion and localized structural defects. Under tensile loading, these inhomogeneities hinder uniform stress distribution, leading to premature failure and reduced tensile performance.

Furthermore, the tensile strength and elongation at break of the PBS/TF/T-PBS composite material gradually increase with the increase in T-PBS viscosity, as shown in Figure 6b. Notably, the composite incorporating T-PBS_4_ demonstrated a 19.7% increase in yield strength and a 78.8% increase in elongation at break compared to the PBS/TF system. Compared to the study by Zoi N. et al. [47], who reinforced PBS with hemp fiber and chaff as fillers, the tensile strength of their product reached a maximum of 9.2% at the same filler loading (15 wt%), which is lower than this study. This enhancement is ascribed to the fact that branched PBS chains significantly improved the interfacial adhesion between TF and the PBS matrix through physical entanglement and polar interactions. Improved interfacial bonding restricted the slippage of TF fibers within the matrix, thereby reducing fiber deformation under external stress. Given that PBS exhibits much higher ductility than TF fibers, strong interfacial adhesion promotes a failure mode transition—from fiber pull-out to fiber fracture—under tensile stress. Figure 6c illustrates the trends in impact and flexural strengths of the composites. Opposite to the continuously increasing tensile performance, both impact and flexural strengths displayed a declining trend with increasing T-PBS viscosity. However, the rate of decline gradually diminished, and the flexural strength of the composites remained higher than that of neat PBS, even in the presence of T-PBS. This indicates that despite the reduced impact resistance, the rigidity contributed by TF was effectively retained.

**Figure 7:**
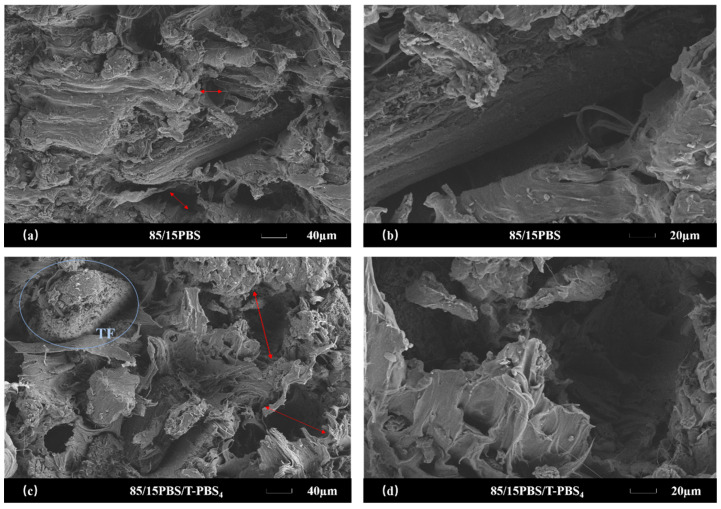
SEM image of the tensile fracture surface of the composite material. (**a**) 85/15PBS at 100 µm; (**b**) enlarged view of holes in 85/15PBS at 20 µm; (**c**) 85/15/T-PBS_4_ at 100 µm; (**d**) enlarged view of holes in 85/15/T-PBS_4_ at 20 µm.

To more intuitively demonstrate the improvement mechanism of the tensile properties of the composite material, Figure 7 shows a SEM comparison of the tensile cross-sections of the 85/15/T-PBS_4_ composite material which showed the best improvement in tensile properties, and the 85/15 composite material without T-PBS. For the composites without T-PBS (85/15PBS), the fiber pull-out holes appeared smooth, indicating weak interfacial bonding. In contrast, the addition of T-PBS led to visibly rougher fracture surfaces, with enlarged pull-out holes and some TF fibers firmly embedded in the matrix, reflecting stronger interfacial adhesion.

### 3.5. Rheological Behavior

**Figure 8:**
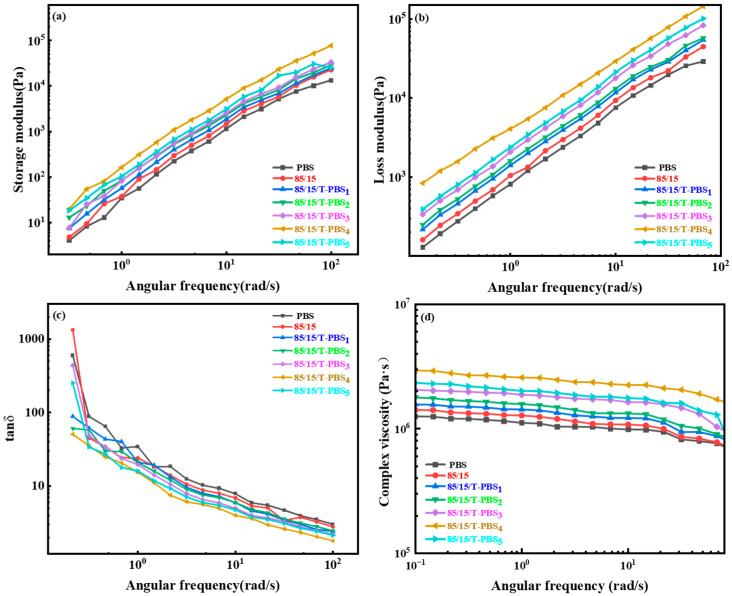
Rheological curves of PBS composite materials. (**a**) storage modulus; (**b**) loss modulus; (**c**) tan δ; (**d**) complex viscosity.

In order to investigate the effect of branching on the melt strength of PBS and PBS-based composites, rotational rheology tests [48] were performed for the polymers, and results are shown in Figure 8. The relationship between the energy storage modulus (G′), loss modulus (G″), tanδ, and angular frequency (ω) were systematically studied. As evident from Figure 8a,b, G′ and G″ are significantly enhanced after the addition of TF and further amplified by branching modification. The increase in G′ indicates improved elastic response due to the formation of a denser physical network, while the increase in G″ demonstrates stronger energy dissipation capacity. Notably, Figure 8c shows the variation in the ratio of G′/G″ (tan δ). For linear PBS, the value of tan δ decreases rapidly with increasing frequency, indicating the typical terminal flow behaviors of melt-like materials with linear structure [49]. With the introduction of T-PBS, the tan δ value decreased significantly, and it was much lower than that of linear PBS [43]. It has been ascribed to the grafting of long-chain branches on the polymer backbone which increases terminal relaxation times [50]. As evident from Figure 8d, the 85/15PBS composite exhibits higher viscosity than PBS, particularly in the low-frequency region, which can be attributed to the interfacial interactions between PBS chains and the hydroxyl groups of TF. These interactions enhance chain entanglement and restrict molecular mobility, thereby intensifying shear-thinning behavior. After the introduction of T-PBS, a relatively higher complex viscosity can be obtained. With the increase in ω, disentanglement between macromolecule chains occurs. Therefore, the amount of entanglement points decreases, resulting in the shear thinning, which is common for pseudoplastic fluids [51]. These results indicate that the combined effect of the branched polymer structure and fiber reinforcement effectively enhanced the rheological performance of the PBS matrix. This improvement is beneficial for regulating flow behavior during processing. As the melt strength and viscosity of composite materials increase, the likelihood of material overflow during injection molding can be reduced, and dimensional stability in the process can be enhanced.

## 4. Conclusions

In this study, a high-performance and fully biodegradable PBS-based composite was successfully developed for the first time, using taxus residue (TF) as a reinforcing filler and branched PBS (T-PBS) as a compatibilizer. T-PBS was synthesized via esterification and polycondensation reactions using succinic acid and 1,4-butanediol as monomers and trimethylolpropane (TMP) as a branching agent. The branched structure significantly improved the interfacial compatibility between TF and the PBS matrix through physical chain entanglement and polar interactions. The incorporation of T-PBS effectively overcame the reduction in yield strength and elongation at break observed in uncompatibilized PBS/TF composites. With increasing T-PBS viscosity, both yield strength and elongation at break of the composites initially increased and then stabilized. In particular, the yield strength and elongation at break were enhanced by 19.7% and 78.8%, respectively, compared to the PBS/TF system without compatibilization. The branching points within T-PBS acted as efficient heterogeneous nucleation centers, thereby promoting crystallization in the composites. Additionally, the branched architecture introduced more molecular entanglements and physical crosslinking sites. This led to improved melt stability and processability of the composite materials.

This work successfully valorized taxus residue, a waste biomass derived from paclitaxel extraction, by transforming it into an effective reinforcing phase for PBS composites. The use of bio-based and biodegradable T-PBS as a compatibilizer avoids the environmental drawbacks associated with conventional, non-eco-friendly compatibilizers, aligning with the principles of sustainable and green composite development. The application of branched PBS as a compatibilizer significantly enhanced the interfacial compatibility, mechanical performance (especially yield strength and elongation at Break), crystallization behavior, and high-temperature thermal stability of PBS/TF composites, while also regulating their rheological properties. These findings offer a promising strategy and technical pathway for the development of high-performance and fully biodegradable PBS composites based on agricultural or industrial biomass waste. Such materials hold great potential for use in packaging, disposable products, automotive interior components, and prospective biomedical applications. Future research may focus on precise tailoring of the T-PBS branching architecture and long-term investigations into the composites’ biodegradation behavior and bioactivity.

## Figures and Tables

**Table 1:** Synthetic preparations of PBS and T-PBS.

Sample	SA/mol	BDO/mol	TMP/%mol	Time/h
PBS	0.5	1.0	-	2
T-PBS_1_	0.5	1.0	0.25	0.5
T-PBS_2_	0.5	1.0	0.25	1
T-PBS_3_	0.5	1.0	0.25	1.5
T-PBS_4_	0.5	1.0	0.25	2
T-PBS_5_	0.5	1.0	0.25	2.5

**Table 2:** Content of PBS-based composite materials.

Sample	PBS/wt%	TF/wt%	T-PBS_1_/wt%	T-PBS_2_/wt%	T-PBS_3_/wt%	T-PBS_4_/wt%	TPBS_5_/wt%
PBS	100	-	-	-	-	-	-
85/15PBS	85	15	-	-	-	-	-
85/15/T-PBS_1_	85	15	8	-	-	-	-
85/15/T-PBS_2_	85	15	-	8	-	-	-
85/15/T-PBS_3_	85	15	-	-	8	-	-
85/15/T-PBS_4_	85	15	-	-	-	8	-
85/15/T-PBS_5_	85	15	-	-	-	-	8

**Table 3:** Viscosity of PBS and T-PBS.

Sample	[η] (dL/g) ^a^
PBS	1.72
T-PBS_1_	0.21
T-PBS_2_	0.78
T-PBS_3_	1.34
T-PBS_4_	1.70
T-PBS_5_	1.85

^a^: Intrinsic viscosity measured using tetrachloroethane/phenol (1/1, m/m) as solvent.

**Table 4:** Thermal stability and thermal performance parameters of PBS and composite materials.

Sample	*T*_m_/°C	*T*_c_/°C	∆*H*_m_/(J·g^−1^)	∆*H*_C_/(J·g^−1^)	*X*_c_/%	*X*_c*_ */%*	*T*_5%_/°C	*T*_85%_/°C	*T*_max_/°C
PBS	114.5	80.8	43.73	45.08	39.6	32.3	325.65	400.16	388.2
85/15PBS	114.8	79.7	59.35	61.08	53.8	43.4	292.63	403.44	382.6
85/15/T-PBS_1_	115.5	84.4	54.22	65.19	49.2	38.8	291.63	401.98	384.0
85/15/T-PBS_2_	114.9	85.4	51.91	64.80	47.1	35.5	292.90	403.26	384.3
85/15/T-PBS_3_	115.4	82.9	53.27	60.36	48.3	39.4	291.63	404.81	384.1
85/15/T-PBS_4_	115.0	82.8	54.16	60.82	49.1	40.5	291.90	408.19	383.4
85/15/T-PBS_5_	114.6	81.9	56.20	59.99	50.9	41.5	294.27	404.08	383.7

Abbreviations: *X*_c_, crystallinity obtained from DSC; *X*_c*_, crystallinity obtained from XRD; *T*_5%_, temperature at 5% weight loss; *T*_85%_, temperature at 85% weight loss; *T*_max_, temperature at maximum weight loss.

**Table 5:** Mechanical properties of PBS and composite materials.

Sample	Elongation at Break (%)	Yield Strength (MPa)	Flexural Strength (MPa)	Elastic Modulus (MPa)	Impact Strength (J/m)
PBS	246.768	40.028	27.144	797	58.427
85/15PBS	6.222	32.426	37.332	1485	42.955
85/15/T-PBS_1_	7.652	35.192	36.691	1346	40.432
85/15/T-PBS_2_	8.436	36.941	34.686	1224	39.808
85/15/T-PBS_3_	10.076	37.124	30.732	1189	39.691
85/15/T-PBS_4_	11.128	38.813	29.652	1112	39.432
85/15/T-PBS_5_	10.908	37.641	29.160	1089	38.144

## Data Availability

The original contributions presented in this study are included in the article. Further inquiries can be directed to the corresponding author.
